# Doppler Shift Tolerance of Typical Pseudorandom Binary Sequences in PMCW Radar

**DOI:** 10.3390/s22093212

**Published:** 2022-04-22

**Authors:** Lucas Giroto de Oliveira, Theresa Antes, Benjamin Nuss, Elizabeth Bekker, Akanksha Bhutani, Axel Diewald, Mohamad Basim Alabd, Yueheng Li, Mario Pauli, Thomas Zwick

**Affiliations:** Institute of Radio Frequency Engineering and Electronics (IHE), Karlsruhe Institute of Technology (KIT), 76131 Karlsruhe, Germany; theresa.antes@kit.edu (T.A.); benjamin.nuss@kit.edu (B.N.); elizabeth.bekker@kit.edu (E.B.); akanksha.bhutani@kit.edu (A.B.); axel.diewald@kit.edu (A.D.); mohamad.alabd@kit.edu (M.B.A.); yueheng.li@kit.edu (Y.L.); mario.pauli@kit.edu (M.P.); thomas.zwick@kit.edu (T.Z.)

**Keywords:** Doppler shift, periodic autocorrelation function, phase-modulated continuous wave, pseudorandom sequence, radar

## Abstract

In the context of all-digital radar systems, phase-modulated continuous wave (PMCW) based on pseudorandom binary sequences (PRBSs) appears to be a prominent candidate modulation scheme for applications such as autonomous driving. Among the reasons for its candidacy are its simplified transmitter architecture and lower linearity requirements (e.g., compared to orthogonal-frequency division multiplexing radars), as well as its high velocity unambiguity and multiple-input multiple-output operation capability, all of which are characteristic of digital radars. For appropriate operation of a PMCW radar, choosing a PRBS whose periodic autocorrelation function (PACF) has low sidelobes and high robustness to Doppler shifts is paramount. In this sense, this article performs an analysis of Doppler shift tolerance of the PACFs of typically adopted PRBSs in PMCW radar systems supported by simulation and measurement results. To accurately measure the Doppler-shift-induced degradation of PACFs, peak power loss ratio (PPLR), peak sidelobe level ratio (PSLR), and integrated-sidelobe level ratio (ISLR) were used as metrics. Furthermore, to account for effects on targets whose ranges are not multiples of the range resolution, oversampled PACFs are analyzed.

## 1. Introduction

Also known as code-modulated, spread spectrum or pseudonoise (PN) radars, phase-modulated continuous-wave (PMCW) radars [1,2,3] are a type of digital radar system that relies on the use of pseudorandom sequences with good autocorrelation properties for yielding minimally biased range profiles. In recent literature, PMCW has also been investigated as a potential modulation scheme for joint radar-communication (RadCom) systems [4,5,6,7,8], which can, e.g., be used for enabling coordination and interference avoidance between radar sensors in highly populated scenarios.

Although inherently high data rates to digital radar systems based on modulation schemes such as orthogonal frequency-division multiplexing (OFDM) [9,10] and orthogonal chirp-division multiplexing (OCDM) [11,12] are also an issue in PMCW [13], the overall system complexity and efficiency of PMCW-based radar systems can be improved with respect to other digital radar modulation schemes if a pseudorandom binary sequence (PRBS) is adopted as a base radar signal. The main advantages of the use of PRBSs comprise the lower linearity requirements and higher efficiency of power amplifiers (PAs) due to the possibility of operating near saturation since a continuous wave (CW)-like signal is transmitted [14], the lack of need for I/Q modulation at the transmitter, and the possibility to use linear-feedback shift registers (LFSRs) instead of digital-to-analog converters (DACs) if PRBSs such as m-sequences are adopted. Furthermore, PRBS-based PMCW radar systems have also been proven to be capable of multiple-input multiple-output (MIMO) operation for direction of arrival (DoA) estimation, which is paramount in applications such as highly automated driving (HAD). In this context, orthogonal binary signals, which can either be orthogonal PRBSs in the cross-correlation sense [8,15,16,17] or outer-coded versions of the same PRBS [8,15,16,18], are assigned to distinct transmit channels and their reflections off targets are received at distinct receive channels and further processed to ultimately enable DoA estimation, e.g., via Fourier beamforming [19,20].

Regardless of whether single-input single-output (SISO) or MIMO operation is aimed, the adopted PRBS by a PMCW radar system should ideally yield an autocorrelation function with low sidelobes and negligible main lobe power degradation under influence of Doppler shifts, so that both erroneous interpretation of sidelobes as targets and signal-to-noise ratio (SNR) loss are avoided. Recent studies in the literature include, e.g., investigation of the sidelobe degradation of the autocorrelation functions of some of the typically used PRBSs in PMCW radars [17] as well as the design of sufficiently long PRBSs to jointly enable a high unambiguous range and yield low sidelobe level [21]. Whereas the first study only considers a limited number of typical PRBSs and the second one does not perform a Doppler shift tolerance analysis, both only evaluate the discrete autocorrelation functions of PRBSs. Although this may provide some degree of information on the distortion of range profiles caused by sidelobes of the autocorrelation functions, it fails to provide information on sidelobes associated with targets whose range is not an integer multiple of the radar range resolution and therefore prevents a fair comparison among potential PRBSs to be used as a base PMCW signal. Similarly to what is done in the context of OFDM radars in [22], a solution to this is to evaluate the oversampled autocorrelation functions of PRBSs of interest, which can be alternatively interpreted as range ambiguity functions and presents a sinc-like shape due to the band-limited nature of the ultimately received PRBS that is processed for range estimation at the receiver side of the PMCW radar system. In this sense, this article performs an analysis of the Doppler shift tolerance of the oversampled periodic autocorrelation functions (PACFs) of typically used PRBSs in PMCW radar systems.

The main contributions of this article are summarized as follows:

- An overview of typically adopted PRBSs in PMCW radar systems, namely m-sequences, Gold sequences, Kasami sequences, almost perfect autocorrelation sequences (APASs), zero correlation zone (ZCZ) sequences, and Golay sequences, and their implications on range and velocity ambiguities for different PRBS lengths. For the aforementioned analyses, an automotive (1GHz bandwidth and 79GHz carrier frequency) and a gesture recognition application (12.5GHz bandwidth and 140GHz carrier frequency) were considered. For the Golay sequences, both the use of complementary pairs and the use of a single Golay sequence as a variation of ZCZ sequences, which to the best of the authors’ knowledge has not been previously reported in PMCW radar literature, are adressed.
- An analysis of the Doppler shift tolerance of the oversampled PACFs of the aforementioned PRBSs for different sequence lengths. For this analysis, a normalized Doppler shift parameter introduced in a previous article [23] was adopted and sequences that achieve similar maximum unambiguous range were compared by using peak power loss ratio (PPLR), peak sidelobe level ratio (PSLR), and integrated-sidelobe level ratio (ISLR) as metrics.

The remainder of this paper is organized as follows. Section 2 outlines the system model of a SISO-PMCW radar system for a generic PRBS, showing closed-form expressions for multiple stages of the radar signal processing chain and formulating the problem of correlation-based range sensing under Doppler shifts. Next, Section 3 presents metrics for evaluating the distortion of the PACF of a PRBS caused by Doppler shifts, which ultimately yields distorted range profiles. A comparative performance analysis is then carried out in Section 4, where the relevant aspects of the considered SISO-PMCW radar system are assessed and the PPLR, PSLR, and ISLR for typically adopted PRBSs in PMCW radar are evaluated to measure the Doppler shift-induced degradation of range profiles. Finally, concluding remarks are given in Section 5.

## 2. System Model

Let a SISO radar system consist of a full-duplex radio-frequency (RF) device, which is capable of transmitting PMCW signals and receiving echoes off targets. The aforementioned signals consist of a carrier signal modulated by a stream of identical PRBSs, having therefore a so-called constant envelope. In this SISO-PMCW radar system, a carrier frequency $f_{c}$ is considered. Additionally, a sampling rate $F_{s}\in R_{\geq 0}|F_{s}$ and a sampling period $T_{s}\in R_{\geq 0}|T_{s}=1/F_{s}$ are adopted, which respectively correspond to the chip rate and the chip period of the PRBS.

**Figure 1:**
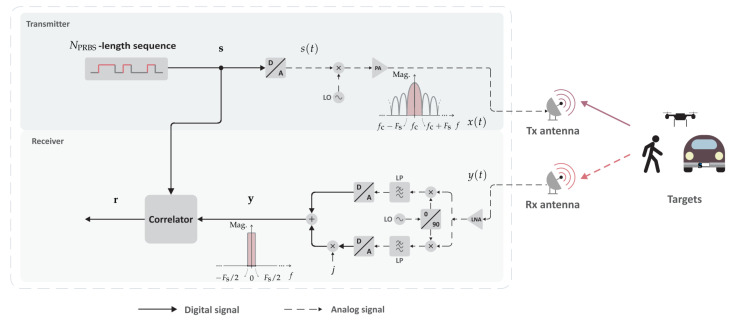
Simplified SISO-PMCW radar processing chain for range estimation.

The processing chain of the considered SISO-PMCW radar system in this study is represented in Figure 1. For the sake of conciseness, the following discussion will be focused on the processing for a single PRBS from the aforementioned stream. At the transmit channel, the PRBS $s\in {\left\{-1,1\right\}}^{N_{\mathrm{PRBS}}\times 1}$ of length $N_{\mathrm{PRBS}}\in N$ is usually generated by an LFSR or DAC. Although the transmission of multiple copies of the PRBS is necessary for Doppler-shift and consequently relative radial velocity estimation, it is henceforth assumed that a single copy of the adopted PRBS is transmitted for the sake of simplicity. The resulting analog signal $s\left(t\right)\in R|\{t\in \left[0,T_{d}\right]\}$ from digital-to-analog (D/A) conversion with sampling rate $F_{s}$ on the PRBS s is then up-converted to the carrier frequency $f_{c}$ and amplified by a power amplifier, becoming $x\left(t\right)\in R|\{t\in \left[0,T_{d}\right]\}$. x(t) is finally transmitted during the dwell time $T_{d}\in R_{\geq 0}|T_{d}=N_{\mathrm{PRBS}}T_{s}$ with power $P_{\mathrm{Tx}}$ by the transmit antenna of gain $G_{\mathrm{Tx}}$. Disregarding spectral sidelobes with lower amplitude, the signal x(t) is assumed to be band-limited to $f\in [f_{c}-F_{s},f_{c}+F_{s}]$, as depicted in Figure 1.

The transmit signal x(t) by the transmit antenna travels through the air at the speed of light in vacuum $c_{0}$, being in sequel reflected off *H* targets toward the SISO-PMCW radar system. Next, the receiver antenna with gain $G_{\mathrm{Rx}}$ receives a signal $y\left(t\right)\in R|\{t\in \left[0,T_{d}\right]\}$ that is attenuated due to path loss and limited radar cross section (RCS) of targets by a factor $\alpha_{h}\in R_{\geq 0}$, and contains information on the range $R_{h}\in R_{\geq 0}$, and the Doppler shift $f_{D,h}\in R$. The latter is specifically related to the target velocity as $f_{D,h}\in R|f_{D,h}=2v_{h}/\lambda_{0}$, where $v_{h}\in R$ denotes the radial velocity of the hth target with respect to the radar and $\lambda_{0}\in R_{\geq 0}|\lambda_{0}=c_{0}/f_{c}$ denoting the free-space wavelength associated with the carrier frequency $f_{c}$.

After amplification by a low-noise amplifier, y(t) is downconverted and low-pass filtered in an I/Q receiver, where it is assumed to be impaired by an additive white Gaussian noise (AWGN) z(t)∈C, prior to analog-to-digital (A/D) conversion with sampling rate $F_{s}$ and combination into real and imaginary parts of the vector $y\in C^{N_{\mathrm{PRBS}}\times 1}$. It is henceforth assumed that spectral sidelobes are filtered out and that the sinc-shaping from the D/A conversion at the transmitter side is compensated for, which results in a band-limited spectrum with $f\in [f_{c}-F_{s}/2,f_{c}+F_{s}/2]$, and therefore a bandwidth $B=F_{s}$, for y as depicted in Figure 1. Therefore, the nth element of y, $n=0,1,\cdots ,N_{\mathrm{PRBS}}-1$, is expressed as

(1) $$y_{n}=\sum_{h=0}^{H-1}\left(\sum_{m=0}^{N_{\mathrm{PRBS}}-1}s_{m}\frac{e^{j2\pi \left[\left(n-m\right)-n_{\Delta ,h}\right]}-1}{e^{j\frac{2\pi }{N}\left[\left(n-m\right)-n_{\Delta ,h}\right]}-1}\right)\alpha_{h}e^{j2\pi f_{D,h}nT_{c}}+z_{n}$$

In this equation, $s_{n}\in \left\{-1,1\right\}$ is the nth element of the PRBS s and $n_{\Delta ,h}=\tau_{h}/T_{c}$, where $\tau_{h}=2R_{h}/c_{0}$ denotes the the round-trip delay associated with the hth target range. The sum over *n* corresponds to the convolution of $s_{n}$ inverse discrete Fourier transform (IDFT) of a discrete-frequency domain vector with unit magnitude response and linear phase defined by the normalized delay $n_{\Delta ,h}$ that $s_{n}$ undergoes. Finally, $z_{n}\in C$ represents the resulting contribution of the AWGN z(t) to $y_{n}$. For the specific case where $n_{\Delta ,h}\in Z$, the aforementioned sum over *n* in (1) becomes a simple convolution of $s_{n}$ with a Kronecker delta. Consequently, (1) can be rewritten as

(2) $$y_{n}=\sum_{h=0}^{H-1}s_{{\langle n-n_{\Delta ,h}\rangle }_{N}}\alpha_{h}e^{j2\pi f_{D,h}nT_{c}}+z_{n}.$$

where ${\langle \cdot \rangle }_{N}$ is the modulo *N* operator. For the sake of simplicity, (2) will be henceforth considered rather than its more generic counterpart (1).

To generate a range profile out of the ultimately received vector y, a periodic cross-correlation with the PRBS s is performed, resulting in the vector $r\in C^{N_{\mathrm{PRBS}}\times 1}$, whose nth element is expressed as

(3) $$r_{n}=\sum_{h=0}^{H-1}\left[\sum_{m=0}^{N_{\mathrm{PRBS}}-1}s_{{\langle m-n_{\Delta ,h}\rangle }_{N}}s_{{\langle -(n-m)\rangle }_{N}}^{*}\right]\alpha_{h}+\sum_{m=0}^{N_{\mathrm{PRBS}}-1}z_{m}s_{{\langle -(n-m)\rangle }_{N}}^{*}.$$

This equation assumes that the effect of Doppler shifts $f_{D,h}$ on the elements of y is negligible except for the introduced phase rotations, which are not shown for the sake of simplicity because a single PRBS is considered. The aforementioned processing is known as pulse compression and artificially compresses the PRBS s into its PACF $R_{ss}\in R^{N_{\mathrm{PRBS}}\times 1}$. Knowing that nth element of $R_{ss}$, $R_{ss,n}\in R$, is expressed as [17]

(4) $$R_{ss,n}=\sum_{m=0}^{N_{\mathrm{PRBS}}-1}s_{m}s_{{\langle -(n-m)\rangle }_{N}}^{*},$$

the resulting nth element of r from pulse compression can be expressed as

(5) $$r_{n}=\sum_{h=0}^{H-1}R_{ss,{\langle n-n_{\Delta ,h}\rangle }_{N}}\alpha_{h}+\sum_{m=0}^{N_{\mathrm{PRBS}}-1}z_{m}s_{{\langle -(n-m)\rangle }_{N}}^{*}.$$

In order for targets ’reflections to be appropriately distinguished from one another and from noise in the range profile r, a number of constraints must be satisfied. Among them are the basic range limitations of the assumed SISO-PMCW radar, which can be derived with a similar analysis to the ones in [8,24]. Those comprise the range resolution $\Delta R=c_{0}/\left(2B\right)$ and the maximum range $R_{\max}=N_{\mathrm{PRBS},\mathrm{usable}}\Delta R=N_{\mathrm{PRBS},\mathrm{usable}}\left[c_{0}/\left(2B\right)\right]$. Although the range resolution is inversely proportional to the frequency bandwidth *B*, the maximum range is jointly limited by the usable length $N_{\mathrm{PRBS},\mathrm{usable}}\in N$ of the PRBS and the range resolution. The usable length $N_{\mathrm{PRBS},\mathrm{usable}}$ is defined as either the unambiguous or low-sidelobe length of the PACF $R_{ss}$ and its relationship with $N_{\mathrm{PRBS}}$ depends on the properties of the adopted PRBSs.

Further constraints on the quality of the range profile r are related to the PACF of the adopted PRBS. To reduce or even avoid distortion of the obtained range profiles, one should adopt a PRBS s that presents an appropriate PACF pattern, with high main lobe level and reasonably low sidelobe level. Moreover, the degradation of the aforementioned PACF pattern by increasing Doppler shifts must be considered. When Doppler shifts increase significantly, the periodic correlation processing stage in Figure 1 previously described by (3) results in

(6) $$r_{n}=\sum_{h=0}^{H-1}\left[\sum_{m=0}^{N_{\mathrm{PRBS}}-1}\left(s_{{\langle m-n_{\Delta ,h}\rangle }_{N}}e^{j2\pi f_{D,h}mT_{c}}\right)s_{{\langle -(n-m)\rangle }_{N}}^{*}\right]\alpha_{h}+\sum_{m=0}^{N_{\mathrm{PRBS}}-1}z_{m}s_{{\langle -(n-m)\rangle }_{N}}^{*}.$$

The pulse compression in this equation can be interpreted as resulting from the periodic cross-correlation function (PCCF) of the pseudorandom sequence s and its Doppler-shifted version or simply as the PACF of s under Doppler shift, which is defined as $R_{s_{D}s}\in R^{N_{\mathrm{PRBS}}\times 1}$ and has its nth element expressed as [17]

(7) $$R_{s_{D}s,n}=\sum_{m=0}^{N_{\mathrm{PRBS}}-1}\left(s_{m}e^{j2\pi f_{D,h}mT_{c}}\right)s_{{\langle -(n-m)\rangle }_{N}}^{*},$$

in which $R_{s_{D}s,n}\in R$ and the index *h* of the radar targets has been omitted for the sake of simplicity. Based on previous studies in the literature, it is expected that the effectively compressed PRBS $R_{s_{D}s}$ will present increasing sidelobe levels along with the Doppler shift $f_{D}$ [17]. In this context, parameters for assessing the degradation of the PACF of typically adopted PRBSs in PMCW radar systems are presented in Section 3.

## 3. Metrics for Assessing Doppler-Shift-Induced Range Profile Distortion

**Figure 2:**
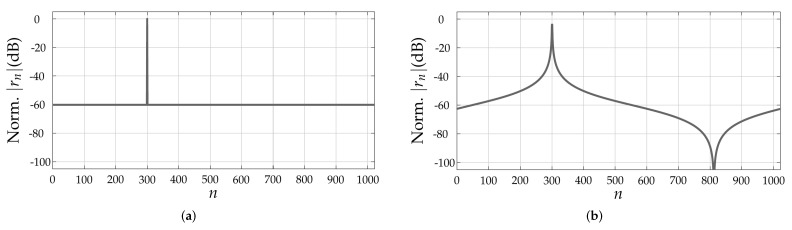
Non-oversampled range profiles obtained in a SISO-PMCW radar system adopting an m-sequence of length $N_{\mathrm{PRBS}}=1023$: (**a**) target at range bin n=300 and (**b**) target at range bin n=300.5.

**Figure 3:**
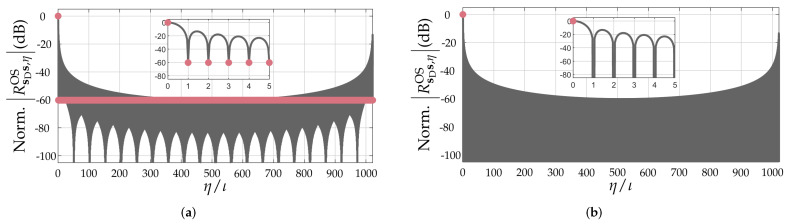
Comparison between Nyquist-sampled (●) and oversampled (**—**, ι=20) PACFs: (**a**) PACF of m-sequence of length $N_{\mathrm{PRBS}}=1023$ and (**b**) ideal PACF of an hypotetical sequence of length $N_{\mathrm{PRBS}}=1023$.

Based on the carried-out discussion in the previous section, it can be concluded that the quality of the range profile r depends on the pattern and Doppler-shift tolerance of the PACF of the adopted PRBS. Although a simple evaluation of the non-oversampled PACFs of PRBSs allows some degree of comparison between different sequences [17], it does not consider the fact that target ranges mostly do not fall onto exact multiples of ΔR, therefore resulting in leakage of the targets’ peaks to neighbor range bins even in non-oversampled range profiles due to the band-limited nature of the PMCW radar system. An example is shown in Figure 2. For this reason, the aforementioned metrics to assess the distortion of the PACF along with increasing Doppler shifts will henceforth assume oversampled PACFs via zero-padding with a factor $\iota \in N_{\geq 0}$ [22]. In this sense, the oversampled PACF of a PRBS s can be denoted by $R_{s_{D}s}^{\mathrm{OS}}\in R^{\iota N_{\mathrm{PRBS}}\times 1}$, whose ηth element is denoted by $R_{s_{D}s,\eta }^{\mathrm{OS}}\in R$, $\eta =0,1,\ldots ,\iota N_{\mathrm{PRBS}}-1$. As an example, Figure 3 compares the critically sampled and the oversampled PACFs for an m-sequence (Figure 3a) and for an ideal, hypothetical sequence (Figure 3b), both with length $N_{\mathrm{PRBS}}=1023$. The aforementioned ideal sequence was designed in the discrete-frequency domain, where both a flat magnitude response and a zero phase were ensured. This design leads to a Kronecker delta non-oversampled PACF and to the sinc-shaped oversampled PACF shown in Figure 3b.

Parameters that can be used to compare the oversampled PACFs of different sequences under Doppler shifts are the PSLR and ISLR [22], which are respectively defined as

(8) $$\mathrm{PSLR}≜\frac{\max_{\eta \in N_{\mathrm{SL}}}\left|R_{s_{D}s,\eta }^{\mathrm{OS}}\right|}{|R_{s_{D}s,0}^{\mathrm{OS}}|}$$

and

(9) $$\mathrm{ISLR}≜\frac{\sum_{\eta \in N_{\mathrm{SL}}}\left|R_{s_{D}s,\eta }^{\mathrm{OS}}\right|}{\sum_{\eta \in N_{\mathrm{ML}}}\left|R_{s_{D}s,\eta }^{\mathrm{OS}}\right|},$$

with $\eta \in N_{\mathrm{SL}}=\left\{\iota \leq \eta \leq \iota N_{\mathrm{PRBS},\mathrm{usable}}-1\right\}\cup \left\{\iota N_{\mathrm{PRBS}}-\iota N_{\mathrm{PRBS},\mathrm{usable}}+1\leq \eta \leq \iota N_{\mathrm{PRBS}}-\iota \right\}$ and $\eta \in N_{\mathrm{ML}}=\left\{0\leq \eta \leq \iota -1\right\}\cup \left\{\iota N_{\mathrm{PRBS}}-\iota +1\leq \eta \leq \iota N_{\mathrm{PRBS}}-1\right\}$ denoting the η intervals for the sidelobes and the mainlobe, respectively. The considered intervals for η are chosen so that two extreme cases are considered, namely, the influence of the sidelobes of eventual targets at range 0 and another target at the maximum range $R_{\max}=N_{\mathrm{PRBS},\mathrm{usable}}\Delta R$ on one another are assessed. Additionally, an increasing main lobe power reduction along with $f_{D}$ is also expected, which can be quantified by the PPLR as [17]

(10) $$\mathrm{PPLR}=\frac{|R_{s_{D}s,0}{|}^{2}}{|R_{ss,0}{|}^{2}}.$$

The mainlobe power reduction measured by the PPLR can be interpreted as an SNR loss or a reduction of the processing gain of $10\log_{10}\left(N_{\mathrm{PRBS}}\right)\mathrm{dB}$ that is expected from the correlation-based range processing in PMCW radars. Consequently, the expected Doppler shifts and resulting processing gain reduction caused by their associated PPLR values must be taken into consideration when defining the link budget of a PMCW radar system, which can be calculated, e.g., as discussed in [16].

Based on the aforementioned parameters, an analysis of the degradation of main lobe and sidelobe levels of the PACF of typical PRBSs for PMCW radar caused by Doppler shifts can be performed to facilitate the choice of an appropriate PRBS that yields in tolerable distortion of obtained range profile. In this article, such an analysis is presented in Section 4.

## 4. Comparative Performance Analysis

In this section, a comparative analysis of the PACFs of typically used PRBSs in PMCW radar systems is performed based on the introduced performance metrics in Section 3. In this context, Section 4.1 briefly describes the considered PRBSs, and Section 4.2 investigates the potential of range profile distortion of those PRBSs by analyzing their PACFs under increasing Doppler shifts.

### 4.1. Investigated Pseudorandom Binary Sequences

The investigated PRBSs in this article are m-sequences, Gold sequences, Kasami sequences, binary APASs, binary ZCZ sequences, and binary Golay sequences. Those are the most typically used PRBSs in PMCW radar systems, and their most relevant characteristics for the analysis in this article are discussed as follows.

#### 4.1.1. m-Sequences

Also known as maximum-length sequence (MLSs), PN sequences, or pseudorandom sequences, m-sequences [25] are a type of PRBS generated by linearly recursive LFSRs [26] of $N_{\mathrm{bit}}^{\mathrm{MLS}}\in N$ bits. In such LFSRs, every $n_{\mathrm{bit}}^{\mathrm{MLS}}\mathrm{th}$ bit, $n_{\mathrm{bit}}^{\mathrm{MLS}}\in \left\{0,1,\ldots ,N_{\mathrm{bit}}^{\mathrm{MLS}}-1\right\}$, is determined by a linear combination of the previous $n_{\mathrm{bit}}^{\mathrm{MLS}}$ bits, so that the resulting m-sequence has an odd period or length equal to $N_{\mathrm{PRBS}}=2^{N_{\mathrm{bit}}^{\mathrm{MLS}}-1}$. For m-sequences, it holds that the PACF usable length is equal to the PRBS length, i.e., $N_{\mathrm{PRBS},\mathrm{usable}}=N_{\mathrm{PRBS}}$.

#### 4.1.2. Gold and Kasami Sequences

Gold sequences [27,28] and Kasami sequences [29] are both PRBSs derived from m-sequences and therefore have length $N_{\mathrm{PRBS}}=2^{N_{\mathrm{bit}}^{\mathrm{MLS}}-1}$. Gold or Kasami sequences are generated in larger sets of orthogonal sequences than achievable sets of orthogonal m-sequences, being therefore usually used in code-division multiplexing (CDM) and code-division multiple access (CDMA) applications, such as MIMO operation of PMCW radar systems [17]. This, however, comes at the cost of increased sidelobe level in their PACFs, which will be investigated in further detail for a SISO-PMCW radar system in Section 4.2. Although relevant, the suitability of sets of orthogonal Gold and Kasami sequences for MIMO operation is left for a future study. Regarding PACF usable length, $N_{\mathrm{PRBS},\mathrm{usable}}=N_{\mathrm{PRBS}}$ is assumed for Gold and Kasami sequences because the sidelobe level of their non-oversampled PACFs is homogeneous.

#### 4.1.3. Almost Perfect Autocorrelation Sequences

Binary APASs are PRBSs whose non-oversmapled PACFs are nearly ideal in the absence of Doppler shifts. In this article, APASs based on p-ary m-sequences [30] are considered [31]. Such APASs have a strong main lobe at n=0 and null sidelobes, with the exception of an additional peak at $n=N_{\mathrm{PRBS}}/2$, having therefore usable PACF length $n=N_{\mathrm{PRBS},\mathrm{usable}}=N_{\mathrm{PRBS}}/2-1$. Additionally, the considered APASs have length $N_{\mathrm{PRBS}}$ that is a multiple of 4 and satisfies the constraint of $N_{\mathrm{PRBS}}/2-1$ being a prime power [17,32].

#### 4.1.4. Zero Correlation Zone Sequences

Similarly to APASs, binary ZCZ sequences are PRBSs whose non-oversampled PACFs are only ideal within a limited interval of samples in the absence of Doppler shifts, which defines its usable length $N_{\mathrm{PRBS},\mathrm{usable}}$. The main difference is that, instead of having only a single extra peak, the PACF of ZCZ sequences present a wider interval with non-zero sidelobes. In this article, the considered ZCZ sequences are assumed to be generated by the algorithm from [33], which provides the highest flexibility regarding PRBS length $N_{\mathrm{PRBS}}$, PACF usable length $N_{\mathrm{PRBS},\mathrm{usable}}$ and number of different sequences of same length [17], the latter being only relevant for MIMO applications.

#### 4.1.5. Golay Sequences

Binary Golay sequences are defined in complementary pairs [34], whose sum of non-oversampled PACFs yields an ideal non-oversampled PACF in the absence of Doppler shifts. In this article, those complementary sequences are henceforth called Golay A and Golay B sequence. In order for Golay sequences A and B to be separately transmitted, a multiplexing strategy has to be chosen, being the most common one time-division multiplexing (TDM). Although CDM, e.g., with Hadamard codes, could be used and still keep the aforementioned complementary sequences binary, it would result in an even longer measurement time [8,16,23]. To avoid mutual interference between those sequences, it is henceforth assumed that cyclic prefix (CPs) of length $N_{\mathrm{CP}}^{\mathrm{Golay}}$ are pretended to each of them before their successive transmission. Assuming an ideal radar channel, each of the received complementary sequences is correlated with their corresponding transmit counterparts at the receiver side of the SISO-PMCW radar system after CP has been removed. The two resulting PACFs are then summed to yield an ultimate PACF. Although the resulting PACF usable length could be equal to $N_{\mathrm{PRBS}}$ due to the expected null sidelobes, the maximum unambiguous range of the SISO-PMCW radar system adopting complementary Golay sequences will be mostly constrained by the CP length $N_{\mathrm{CP}}^{\mathrm{Golay}}$ that is appended to each of the complementary sequences. Consequently, the usable length of PACF resulting from the sum of the PACFs of the complementary Golay sequences A and B is $N_{\mathrm{PRBS},\mathrm{usable}}=N_{\mathrm{CP}}^{\mathrm{Golay}}$. In this article, CPs of length $N_{\mathrm{CP}}^{\mathrm{Golay}}=N_{\mathrm{PRBS}}$ will be henceforth assumed for each of the complementary sequences so that no reduction in $N_{\mathrm{PRBS},\mathrm{usable}}$ is experienced. Another possibility for binary Golay sequences is to transmit just one of the sequences from the complementary pair, i.e., Golay A or Golay B, and use it as a ZCZ sequence. In this context, PACFs with maximum usable length $N_{\mathrm{PRBS},\mathrm{usable}}<N_{\mathrm{PRBS}}$ are observed.

#### 4.1.6. Parameterization Examples for the Investigated Pseudorandom Binary Sequences

**Figure 4:**
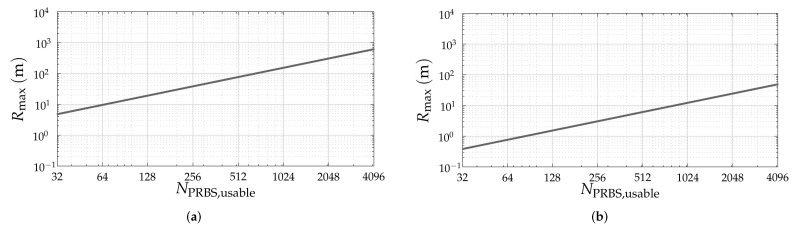
Maximum unambiguous range $R_{\max}=N_{\mathrm{PRBS},\mathrm{usable}}\Delta R$ for the considered (**a**) automotive and (**b**) gesture recognition scenarios.

Given the investigated PRBSs, Table 1 lists the considered PRBS lengths $N_{\mathrm{PRBS}}$ and PRBS usable lengths $N_{\mathrm{PRBS},\mathrm{usable}}$ in this article. For all investigated PRBSs, lenghts of approximately $N_{\mathrm{PRBS}}\in \left\{256,512,1024,2048,4096\right\}$ have been considered when the PRBS of such length exists. To illustrate the achievable maximum range as a function of $N_{\mathrm{PRBS},\mathrm{usable}}$, two scenarios are considered. The first is aiming automotive applications, where a sampling rate $F_{s}=1G\mathrm{Hz}$ and a carrier frequency $f_{c}=79G\mathrm{Hz}$ are adopted, which results in the range resolution ΔR=0.15m. The second application is gesture recognition, in which $F_{s}=12.5G\mathrm{Hz}$ and $f_{c}=140G\mathrm{Hz}$ are considered and ΔR=0.01m is achieved. The resulting maximum unambiguous ranges for both scenarios as a function of $N_{\mathrm{PRBS},\mathrm{usable}}$ are shown in Figure 4. An analysis of this figure based on the $N_{\mathrm{PRBS}}$ and $N_{\mathrm{PRBS},\mathrm{usable}}$ pairs listed in Table 1 reveals that ZCZ sequences, Golay A and B sequences without sum of their PACFs, and APASs yield the lowest maximum unambiguous range values, in this order, because they have $N_{\mathrm{PRBS},\mathrm{usable}}<N_{\mathrm{PRBS}}$.

### 4.2. Evaluation of Doppler Shift Distortion of Range Profiles

**Figure 5:**
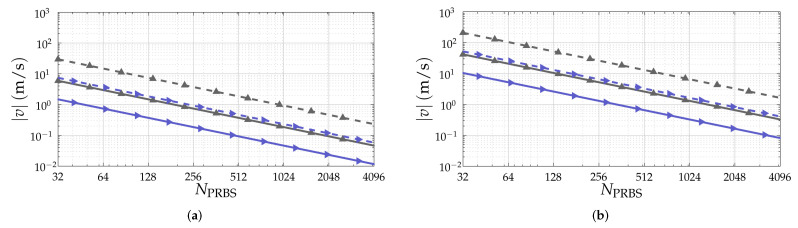
Corresponding relative radial velocity $\left|v\right|=\lambda_{0}\left|f_{D}\right|/2$ to normalized Doppler shifts $\left|f_{D}/\Delta f\right|=0.1$ (**—** and ► for combination of complementary sequences Golay A and B with $N_{\mathrm{CP}}^{\mathrm{Golay}}=N_{\mathrm{PRBS}}$ for each, and **—** and ▲ for remaining PRBSs) and $\left|f_{D}/\Delta f\right|=0.5$ (**– –** and ► for combination of complementary sequences Golay A and B with $N_{\mathrm{CP}}^{\mathrm{Golay}}=N_{\mathrm{PRBS}}$ for each, and **– –** and ▲ for remaining PRBSs) for the considered (**a**) automotive and (**b**) gesture recognition scenarios.

Defining a frequency resolution $\Delta f=F_{s}/N_{\mathrm{PRBS}}$ for the considered SISO-PMCW radar system, which follows the same principle of subcarrier frequency spacing or bandwidth in OFDM-based systems, one can define a normalized Doppler shift parameter as $f_{D}/\Delta f$ [23]. Besides the static case, where $f_{D}/\Delta f=0$ is experienced, two important points in the $f_{D}/\Delta f$ axis are $\left|f_{D}/\Delta f\right|=0.1$, which is usually assumed as the maximum tolerable normalized Doppler shift in OFDM-based radar and communication systems [24,35], and $\left|f_{D}/\Delta f\right|=0.5$, which is associated with the maximum unambiguous velocity of the considered SISO-PMCW radar system given by $\left|v_{\max,\mathrm{unamb}}\right|=\left(0.5\Delta f\right)c_{0}/\left(2f_{c}\right)$. Considering the same parameters of the automotive and gesture recognition scenarios mentioned in Section 4.1, Figure 5 shows the corresponding relative radial velocities to the aforementioned $\left|f_{D}/\Delta f\right|$ values as a function of $N_{\mathrm{PRBS}}$. In this figure, lower relative radial velocities are observed for the combination of the complementary sequences Golay A and B due to the overall transmission time for the samples associated with a single range profile. Because $N_{\mathrm{CP}}^{\mathrm{Golay}}=N_{\mathrm{PRBS}}$ is adopted for both complementary sequences of length $N_{\mathrm{PRBS}}$, this time becomes $T_{d}=\left(2N_{\mathrm{PRBS}}+2N_{\mathrm{CP}}^{\mathrm{Golay}}\right)T_{s}=4N_{\mathrm{PRBS}}T_{s}$. Based on the aforementioned normalized Doppler shift parameter, a comparative analysis of the Doppler shift tolerance of PRBSs of different lengths is performed as follows.

**Figure 6:**
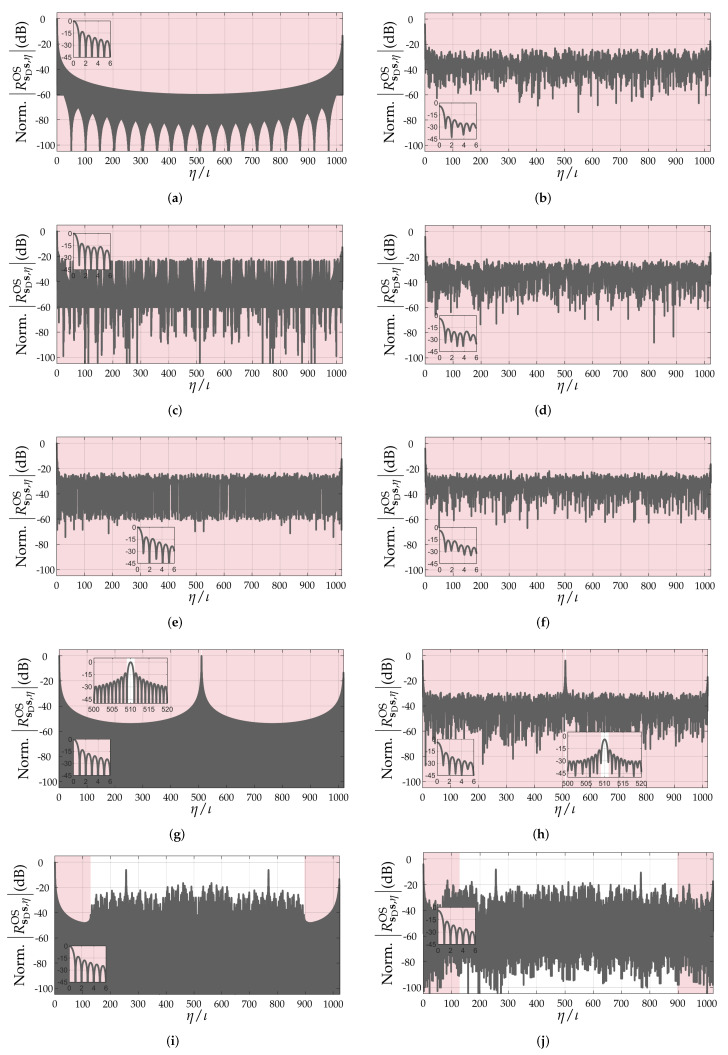
Normalized oversampled PACFs (ι=20) under $f_{D}/\Delta f=0$ and $\left|f_{D}/\Delta f\right|=0.5$ of (**a**,**b**) m-sequence, (**c**,**d**) Gold sequence, (**e**,**f**) Kasami sequence, (**g**,**h**) APAS, and (**i**,**j**) ZCZ sequence. An with approximate length of $N_{\mathrm{PRBS}}=1024$ was adopted for all PRBSs. The relevant PACF intervals described in Section 3 are highlighted in red.

**Figure 7:**
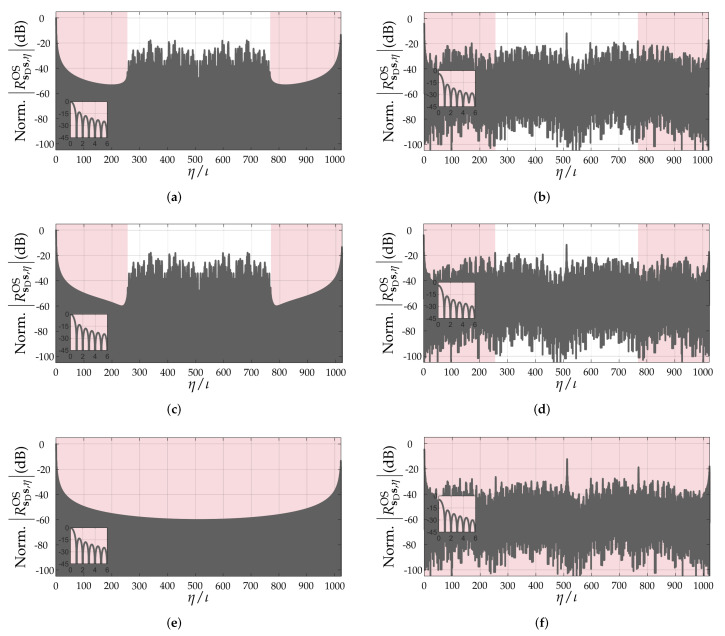
Normalized oversampled PACFs (ι=20) under $f_{D}/\Delta f=0$ and $\left|f_{D}/\Delta f\right|=0.5$ of Golay sequences (**a**,**b**) A and (**c**,**d**) B acting as ZCZ sequences, and (**e**,**f**) sum of the PACFs of the complementary Golay sequences A and B. An with approximate length of $N_{\mathrm{PRBS}}=1024$ was adopted for all PRBSs. The relevant PACF intervals described in Section 3 are highlighted in red.

To illustrate the effect of Doppler shifts on the PACFs of the considered PRBSs, Figure 6 shows the oversampled PACFs (ι=20) of the PRBSs m-sequence, Gold sequence, Kasami sequence, APAS, and ZCZ with approximate length of $N_{\mathrm{PRBS}}=1024$ under null Doppler shift and at $\left|f_{D}/\Delta f\right|=0.5$. Similarly, Figure 7 shows the same aforementioned PACF results for the Golay sequences A and B both acting as ZCZ sequences and as complementary sequences that are summed. In both figures, the set of all relevant PACF intervals for calculating the PSLR, ISLR, and PPLR metrics, which are described in Section 3, are highlighted in red. In the null Doppler shift cases, it can be observed that ZCZ, APAS, and Golay sequences (both A, B, and their sum) yield sinc-shaped oversampled PACFs in their usable intervals, which is due to the band-limited nature of the considered SISO-PMCW radar system, the remaining sequences yield oversampled PACFs with somewhat different shapes. As for the other PRBSs, m-sequences yield nearly sinc-shaped oversampled PACFs for their whole length, whereas Gold and Kasami sequences present rather irregular sidelobe patterns. When the Doppler shift is increased to $\left|f_{D}/\Delta f\right|=0.5$, all of the considered PRBSs suffer peak power loss and present irregular sidelobe patterns.

To accurately quantify the Doppler shift-induced degradation of the oversampled PACFs for different $N_{\mathrm{PRBS}}$ values, an analysis assuming the described parameters in Section 3 is carried out based on simulation and measurement results. The adopted setup for obtaining the proof-of-concept measurement results consists of a monostatic SISO-PMCW radar supporting all PRBSs listed in Table 1 and the radar target simulator (RTS) described in [36] and used in [37,38,39], both implemented on a Zynq UltraScale+ RFSoC ZCU111 from Xilinx, Inc. To avoid multiple reflections, coaxial cables are used to directly connect the DACs and analog-to-digital converter (ADCs) of the radar connected to the ADCs and DACs of the RTS, respectively. For the aforementioned measurements, a sampling rate of $F_{s}=100M\mathrm{Hz}$ was used for the SISO-PMCW radar system and the aforementioned RTS was used to introduce the Doppler shifts that result in $f_{D}/\Delta f$ values ranging from 0 to 0.5. Although a low sampling rate was adopted to keep the absolute Doppler shifts at reasonable values that can be supported by the RTS, it is recalled that the normalized Doppler shifts $f_{D}/\Delta f$ drive the ultimate degradation of the PACF, which allows for predicton of the behavior of SISO-PMCW radar systems with other $F_{s}$ values based on the presented results.

#### 4.2.1. Peak Power Loss Ratio

**Figure 8:**
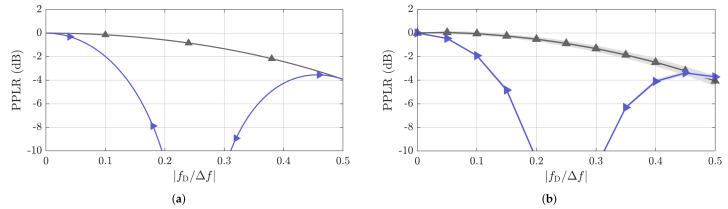
Simulated (**a**) and measured (**b**) PPLR as a function of $f_{D}/\Delta f$. Except for the combination of complementary sequences Golay A and B (►), which achieves the same PPLR profile for all $N_{\mathrm{PRBS}}$ values, all other considered PRBSs listed in Table 1 (▲) achieve the same result. For the measurement results, the mean PPLR values are shown as solid lines, and their standard deviations are indicated by the shading in the background.

The attained PPLR results are shown in Figure 8. Because, except for the sums of the PACFs of the complementary sequences Golay A and B, all considered PRBSs from Table 1 theoretically yield the same PPLR versus $f_{D}/\Delta f$ results as shown in Figure 8a, their measured PPLR values for all considered PRBSs were combined. The same was done for the combination of the complementary sequences Golay A and B, which yields the same PPLR profile regardless of the length $N_{\mathrm{PRBS}}$ of each of the complementary sequences. To account for variations in the measurements, both the mean PPLR (continuous line) and its standard deviation (shading in the background) are shown in Figure 8b. An analysis of the simulated results in Figure 8a and the measured results in Figure 8b show a close match, being the minor deviations explained by imperfect measurement calibration.

The achieved results show a negligible PPLR of only −0.14dB at $\left|f_{D}/\Delta f\right|=0.1$ for the greater set of PRBSs, i.e., all but the combination of the complementary sequences Golay A and B. At $\left|f_{D}/\Delta f\right|=0.5$; however, a PPLR of around −4dB is observed, which causes a more significant reduction of the ideal processing gain of $10\log_{10}\left(N_{\mathrm{PRBS}}\right)\mathrm{dB}$ and ultimately reduces the SNR of target reflections in the range profile. The achieved results can, e.g., be illustrated by the lower normalized mainlobe levels at $\left|f_{D}/\Delta f\right|=0.5$ compared to their original levels at $f_{D}/\Delta f=0$ in the previously discussed Figure 6 and Figure 7 for $N_{\mathrm{PRBS}}≊1024$.

As for the sum of the PACFs of the complementary sequences Golay A and B, much more relevant PPLR degradation is observed, with worse values than −10dB for $\left|f_{D}/\Delta f\right|$ between 0.2 and 0.3. The reason for the PPLR degradation is the Doppler shift-induced phase rotation $e^{j2\pi f_{D}\left(2N_{\mathrm{PRBS}}/F_{s}\right)}=e^{j4\pi (f_{D}/\Delta f)}$ between the evaluated Golay A and B sequences assuming the use of a CP of length $N_{\mathrm{CP}}^{\mathrm{Golay}}=N_{\mathrm{PRBS}}$, which results in a non-coherent accumulation of the PACF of the complementary sequences and, consequently, in both a processing gain and SNR reduction. It is worth highlighting that although using shorter CP lengths would yield less PPLR degradation and higher maximum unambiguous velocity, it would also reduce the maximum unambiguous range. For a more detailed description of the non-coherent accumulation effect, the reader is referred to the analysis in a previous study [23].

#### 4.2.2. Peak-to-Sidelobe Level Ratio

**Figure 9:**
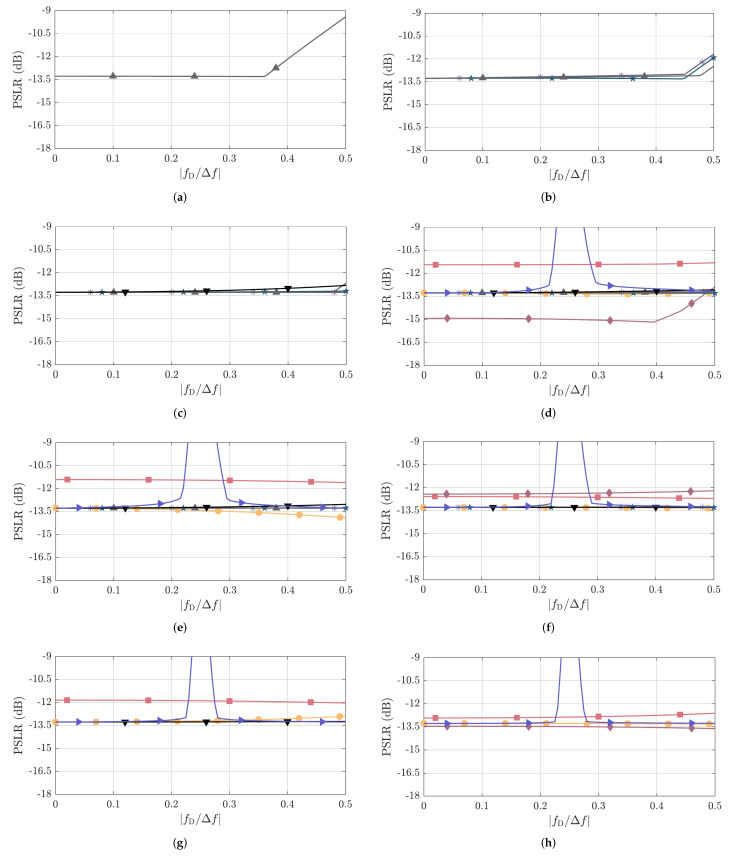
Simulated PSLR as a function of $f_{D}/\Delta f$ for the considered PRBSs with approximate PACF usable length of (**a**) $N_{\mathrm{PRBS},\mathrm{usable}}=32$, (**b**) $N_{\mathrm{PRBS},\mathrm{usable}}=64$, (**c**) $N_{\mathrm{PRBS},\mathrm{usable}}=128$, (**d**) $N_{\mathrm{PRBS},\mathrm{usable}}=256$, (**e**) $N_{\mathrm{PRBS},\mathrm{usable}}=512$, (**f**) $N_{\mathrm{PRBS},\mathrm{usable}}=1024$, (**g**) $N_{\mathrm{PRBS},\mathrm{usable}}=2048$, and (**h**) $N_{\mathrm{PRBS},\mathrm{usable}}=4096$. The considered PRBSs are m-sequence (●), Gold sequence (*■*), Kasami sequence (⧫), APAS (*▼*), ZCZ sequence (▲), Golay A (∗), Golay B (★), and the combination of complementary sequences Golay A and B (►). The correspondence between their $N_{\mathrm{PRBS},\mathrm{usable}}$ and $N_{\mathrm{PRBS}}$ values is listed in Table 1.

**Figure 10:**
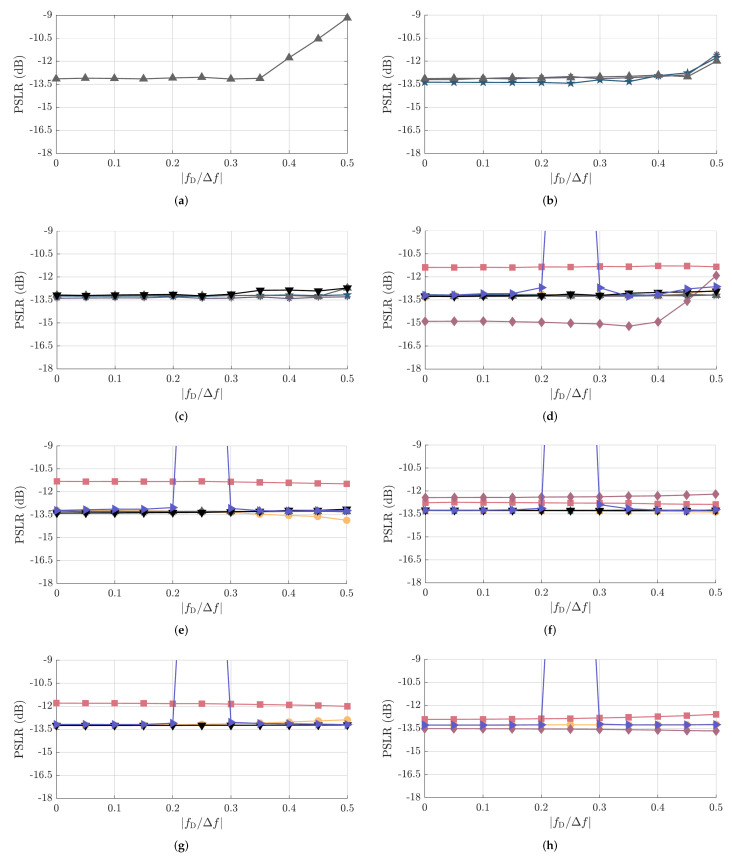
Measured PSLR as a function of $f_{D}/\Delta f$ for the considered PRBSs with approximate PACF usable length of (**a**) $N_{\mathrm{PRBS},\mathrm{usable}}=32$, (**b**) $N_{\mathrm{PRBS},\mathrm{usable}}=64$, (**c**) $N_{\mathrm{PRBS},\mathrm{usable}}=128$, (**d**) $N_{\mathrm{PRBS},\mathrm{usable}}=256$, (**e**) $N_{\mathrm{PRBS},\mathrm{usable}}=512$, (**f**) $N_{\mathrm{PRBS},\mathrm{usable}}=1024$, (**g**) $N_{\mathrm{PRBS},\mathrm{usable}}=2048$, and (**h**) $N_{\mathrm{PRBS},\mathrm{usable}}=4096$. The considered PRBSs are m-sequence (●), Gold sequence (■), Kasami sequence (⧫), APAS (*▼*), ZCZ sequence (▲), Golay A (∗), Golay B (★), and the combination of complementary sequences Golay A and B (►). The correspondence between their $N_{\mathrm{PRBS},\mathrm{usable}}$ and $N_{\mathrm{PRBS}}$ values is listed in Table 1.

Next, the simulated PSLR results for all PRBSs from Table 1 are shown in Figure 9. Those are validated by the measured PSLR results as functions of $f_{D}/\Delta f$ presented in Figure 10 for all considered PRBSs.

Overall, very similar PSLR performance is achieved by most of the considered PRBSs, which also present relatively stable PSLR values over the considered $f_{D}/\Delta f$ range. This can, e.g., be illustrated by the highest sidelobe level in the previously discussed Figure 6 and Figure 7 for $N_{\mathrm{PRBS}}≊1024$. Among the exceptions are the ZCZ sequence with $N_{\mathrm{PRBS}}\in \left\{256,512,1024\right\}$, Golay A and Golay B sequences with $N_{\mathrm{PRBS}}=256$, Kasami sequence with $N_{\mathrm{PRBS}}=255$, and APAS with $N_{\mathrm{PRBS}}=256$, for which the PSLR degrades after reaching high $f_{D}/\Delta f$ values. Among the aforementioned sequences, the ones with lower $N_{\mathrm{PRBS}}$ start suffering from observable PSLR degradation at lower $\left|f_{D}/\Delta f\right|$ values compared to their counterparts with longer PRBS length $N_{\mathrm{PRBS}}$. This can be explained by the fact that the ratio $f_{D}/\Delta f$ corresponds to a shift in samples in the discrete-frequency domain. Therefore, the same shift by $f_{D}/\Delta f$ samples becomes relatively less relevant in comparison to the total number of samples $N_{\mathrm{PRBS}}$ as the PRBS length increases. With the increased relevance of Doppler shifts, the nearest sidelobe to the mainlobe, which is not significantly affected by Doppler shifts as depicted in Figure 6 and Figure 7, does not dominate the PSLR trend anymore. Rather, sidelobes located far from the mainlobe may become stronger than the aforementioned one and degrade the PSLR performance. Further exceptions are the combinations of the complementary sequences Golay A and B, which tend to behave similarly as the remaining PRBSs of similar $N_{\mathrm{PRBS},\mathrm{usable}}$ for low and high $\left|f_{D}/\Delta f\right|$, but suffer severe PSLR degradation in the middle range of the considered $\left|f_{D}/\Delta f\right|$ values (between 0.2 and 0.3, approximately) due to the mainlobe degradation effect described in Section 4.2.1.

Focusing on $\left|f_{D}/\Delta f\right|\leq 0.1$, which is typically assumed, e.g., in OFDM radar systems, it is seen that all considered PRBSs experience negligible PSLR degradation and most of them present nearly the same PSLR of around −13.27dB, which is related to the sinc-like shape of the oversampled PACFs. Among the exceptions are the Gold sequences, which present PSLRs approximately 0.36dB to 1.87dB higher than the aforementioned −13.27dB depending on the sequence length, and Kasami sequences, whose variants with $N_{\mathrm{PRBS}}=255$, $N_{\mathrm{PRBS}}=1023$, and $N_{\mathrm{PRBS}}=4095$ present a PPLR 1.65dB lower, 0.86dB higher, and 0.21dB lower than the trend for the remaining sequences of achieving similar $N_{\mathrm{PRBS},\mathrm{usable}}$, respectively.

#### 4.2.3. Integrated-Sidelobe Level Ratio

Unlike in the PPLR and PSLR results, where nearly all PRBSs perform equally and only small differences are observed, more relevant changes in the ISLR are observed due to the fact that it considers the whole range of sidelobes associated with $N_{\mathrm{PRBS},\mathrm{usable}}$ as described in Section 3. This is observed, e.g., in Figure 6 and Figure 7 for $N_{\mathrm{PRBS}}≊1024$, where significant differences in the degradation of the sidelobe patterns of the PACFs of different PRBSs are observed at $\left|f_{D}/\Delta f\right|=0.5$ w.r.t. their counterparts at $\left|f_{D}/\Delta f\right|=0$.

**Figure 11:**
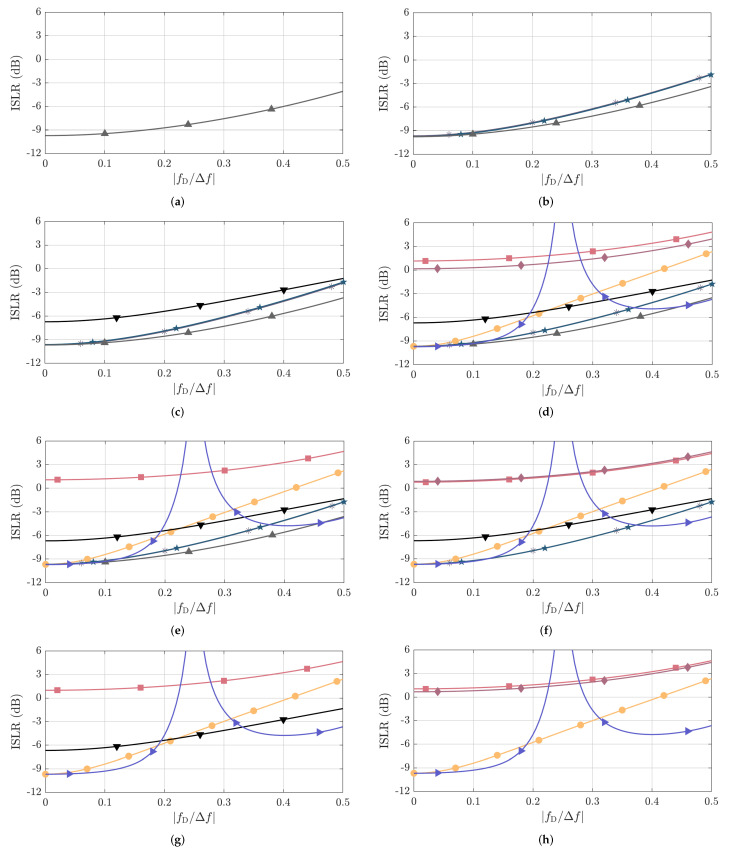
Simulated ISLR as a function of $f_{D}/\Delta f$ for the considered PRBSs with approximate PACF usable length of (**a**) $N_{\mathrm{PRBS},\mathrm{usable}}=32$, (**b**) $N_{\mathrm{PRBS},\mathrm{usable}}=64$, (**c**) $N_{\mathrm{PRBS},\mathrm{usable}}=128$, (**d**) $N_{\mathrm{PRBS},\mathrm{usable}}=256$, (**e**) $N_{\mathrm{PRBS},\mathrm{usable}}=512$, (**f**) $N_{\mathrm{PRBS},\mathrm{usable}}=1024$, (**g**) $N_{\mathrm{PRBS},\mathrm{usable}}=2048$, and (**h**) $N_{\mathrm{PRBS},\mathrm{usable}}=4096$. The considered PRBSs are m-sequence (●), Gold sequence (■), Kasami sequence (⧫), APAS (*▼*), ZCZ sequence (▲), Golay A (∗), Golay B (★), and the combination of complementary sequences Golay A and B (►). The correspondence between their $N_{\mathrm{PRBS},\mathrm{usable}}$ and $N_{\mathrm{PRBS}}$ values is listed in Table 1.

**Figure 12:**
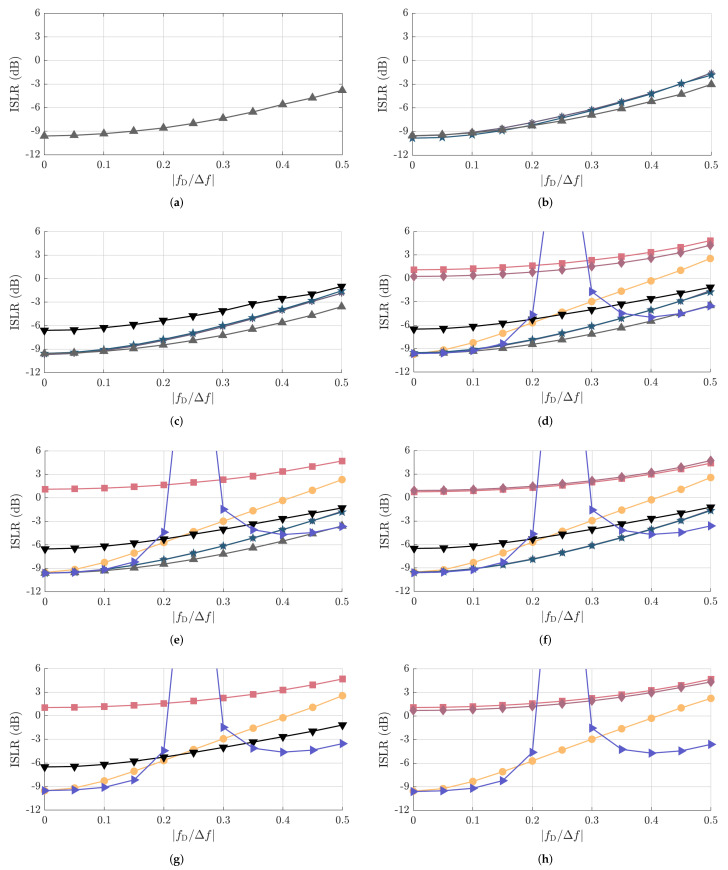
Measured ISLR as a function of $f_{D}/\Delta f$ for the considered PRBSs with approximate PACF usable length of (**a**) $N_{\mathrm{PRBS},\mathrm{usable}}=32$, (**b**) $N_{\mathrm{PRBS},\mathrm{usable}}=64$, (**c**) $N_{\mathrm{PRBS},\mathrm{usable}}=128$, (**d**) $N_{\mathrm{PRBS},\mathrm{usable}}=256$, (**e**) $N_{\mathrm{PRBS},\mathrm{usable}}=512$, (**f**) $N_{\mathrm{PRBS},\mathrm{usable}}=1024$, (**g**) $N_{\mathrm{PRBS},\mathrm{usable}}=2048$, and (**h**) $N_{\mathrm{PRBS},\mathrm{usable}}=4096$. The considered PRBSs are m-sequence (●), Gold sequence (■), Kasami sequence (⧫), APAS (*▼*), ZCZ sequence (▲), Golay A (∗), Golay B (★), and combination of complementary sequences Golay A and B (►). The correspondence between their $N_{\mathrm{PRBS},\mathrm{usable}}$ and $N_{\mathrm{PRBS}}$ values is listed in Table 1.

The simulated and mean measured ISLR results are shown in Figure 11 and Figure 12, respectively, and are in good agreement for all PRBSs from Table 1 and $\left|f_{D}/\Delta f\right|$ values. The achieved results show that the oversampled PACFs of the complementary sequences Golay A and B achieved the highest ISLR values of all for $\left|f_{D}/\Delta f\right|$ between around 0.2 and 0.3, which is due to the increase of the sidelobes that otherwise tends to be similar as in the ZCZ sequences and the reduction of the mainlobe power as described in Section 4.2.1. The next highest ISLR values compared to the remaining sequences of similar $N_{\mathrm{PRBS},\mathrm{usable}}$ over the whole considered $\left|f_{D}/\Delta f\right|$ range are achieved by the oversampled PACFs of Gold and Kasami sequences. Apart from the two aforementioned sequences, APAS present the worst ISLR values for reasonable normalized Doppler shifts, i.e., $\left|f_{D}/\Delta f\right|\leq 0.1$, being followed by m-sequences, Golay A and B sequences, and ZCZ sequences, all with very similar ISLR performances. If, however, higher normalized Doppler shift values are considered, the ISLR associated with oversampled m-sequence PACFs degrades rapidly and tends to yield close values to the ISLRs of the Gold and Kasami sequences. The second most severe ISLR degradation is experienced by Golay A and B sequences, which, however, still achieve ISLR values more than 3dB lower than the ones achieved by m-sequences. Furthermore, APAS and ZCZ experience similar ISLR degradation. Finally, the achieved results show that ZCZ sequences achieve the lowest ISLR over the whole considered $\left|f_{D}/\Delta f\right|$ range compared to the other PRBSs with similar $N_{\mathrm{PRBS},\mathrm{usable}}$ when there exists a ZCZ sequence for the given $N_{\mathrm{PRBS}}$ and $N_{\mathrm{PRBS},\mathrm{usable}}$ pair.

#### 4.2.4. Additional Remarks

Finally, it is worth highlighting that, although the PSLR and ISLR enable an objective comparison among the considered PRBSs, which is the aim of this article, they must be analyzed with care when assessing the overall quality of obtained range profiles. Among the reasons for this are (i) the fact that the nearest sidelobe to the mainlobe dominates the PSLR performance for most considered PRBSs and sequence lengths $N_{\mathrm{PRBS}}$ as discussed in Section 4.2.2, and (ii) the fact that most of the integrated sidelobe level used for ISLR calculation is concentrated around the mainlobe. The aforementioned factors may result in the masking of a significant dynamic range reduction at some regions of the PACF by the PSLR and ISLR metrics. This can be clearly seen, e.g., when observing the Doppler-shift degraded oversampled PACF for Gold sequences in Figure 6d compared to its original form in Figure 6c, which is not reflected by the nearly constant PSLR in Figure 9 and Figure 10 nor by the ISLR degradation of around 3.5dB in Figure 11 and Figure 12. With the use of range windowing functions, which are commonly used in practice but not considered in this article for the sake of conciseness, both PSLR and ISLR metrics tend to better reflect changes in the dynamic range.

## 5. Conclusions

This article presented an analysis of Doppler shift tolerance of the oversampled PACFs of typically adoped PRBSs in PMCW radars, namely m-sequences, Gold sequences, Kasami sequences, APASs, ZCZ sequences, and Golay sequences. After a mathematical formulation of the problem of range estimation via correlation in PMCW radar systems under Doppler shifts, the need for evaluating oversampled PACFs to capture effects on targets that are not at distances that are exact multiples of the range resolution was discussed. Next, PSLR, ISLR, and PPLR parameters were adopted for measuring the Doppler shift-induced degradation of oversampled PACFs of the aforementioned PRBSs and consequently ultimately generated range profiles. Finally, a numerical analysis supported by simulation and measurement results was carried out.

The achieved results showed that, to avoid unwanted high sidelobes, only a short section of the output range profiles from the correlation operation is used in PMCW radar systems based on ZCZ sequences, Golay A and B sequences (used as individual sequences in a similar manner as ZCZ sequences) without sum of their oversampled PACFs, and APASs. Consequently, these PRBSs yield the lowest maximum unambiguous range values compared with the remaining PRBSs of same length. Furthermore, if the oversampled PACFs of complementary Golay sequences are to be summed, then a multiplexing strategy has to be adopted. In this article, TDM was the chosen approach to keep the baseband PMCW signal binary, which leads to a higher transmission time for the samples associated with a single range profile and consequently lower tolerable relative radial velocities.

As for the Doppler shift-induced degradation of oversampled PACFs, it was shown that all sequences yield the same PPLR results. The only exception is the sum of the oversampled PACFs of the complementary sequences Golay A and B, which is severely degraded for certain Doppler shift values due to non-coherent sum or accumulation of the complementary sequences. In terms of PSLR, most sequences present negligible degradation. An exception is the combination of the complementary sequences Golay A and B due to the main lobe degradation observed in the PPLR analysis. Furthermore, ZCZ sequences, Golay A and B sequences used as individual sequences, and Kasami sequences of low PRBS length also present PSLR degradation, which was of less than 4dB for a Doppler shift range that yields relative radial velocities close to the maximum unambiguous one. Finally, the oversampled PACFs of the analyzed PRBSs in this article yielded distinct ISLR values and degradation over the considered Doppler shift range. If, however, only Doppler shift values below the one defined by the same criterion for maximum tolerable velocity in OFDM radars are considered, most PRBSs yield very similar ISLR values and negligible degradation. The only exceptions are APASs, which yield around 3dB higher ISLR, and Gold and Kasami sequences, which yield ISLR values around 9dB higher than observed in the oversampled PACFs of the remaining PRBSs. Consequently, if a hardware-efficient design of a PMCW radar system is sought, m-sequences appear as good candidates due to their good robustness to moderate Doppler shifts defined by the aforementioned criterion originally set for OFDM radars, and their possibility of generation with LFSRs instead of complex high-speed DACs.

## Figures and Tables

**Table 1:** Considered combinations of PRBS lengths $N_{\mathrm{PRBS}}$ and usable PACF lengths $N_{\mathrm{PRBS},\mathrm{usable}}$ for the investigated PRBSs.

Sequence	$N_{\mathrm{PRBS}}$	$N_{\mathrm{PRBS},\mathrm{usable}}$
m-sequence	255	255
	511	511
	1023	1023
	2047	2047
	4095	4095
Gold	255	255
	511	511
	1023	1023
	2047	2047
	4095	4095
Kasami	255	255
	1023	1023
	4095	4095
APAS	256	127
	512	251
	1020	509
	2044	1021
	4008	2003
ZCZ	256	32
	512	64
	1024	128
	2048	256
	4096	512
Golay A or Golay B	256	64
	512	128
	1024	256
	2048	512
	4096	1024
Combined Golay A and B	256	256
	512	512
	1024	1024
	2048	2048
	4096	4096

## Data Availability

Not applicable.
